# Giant Flexoelectro-optic Effect with Liquid Crystal Dimer CB7CB

**DOI:** 10.1038/srep41333

**Published:** 2017-01-24

**Authors:** Andrii Varanytsia, Liang-Chy Chien

**Affiliations:** 1 Liquid Crystal Institute and Chemical Physics Interdisciplinary Program, Kent State University, Kent, Ohio 44242, USA

## Abstract

We demonstrate a giant flexoelectro-optic behavior of liquid crystal dimer CB7CB. Flexoelectric properties of CB7CB experimentally characterized by measured angle of an in-plane rotation of helical axis (HA) in polymer stabilized uniform lying helix cholesteric liquid crystal. The 45° rotation of HA providing full intensity modulation of transmitted through a pair of crossed polarizers light, is achieved with 4.5 V/μm with a sub-millisecond electro-optic switching time. Reported properties enable application of CB7CB in applications of the flexoelectric effect in fast switching photonic and electro-optic devices.

Electro-optic phenomena of liquid crystals (LC) are vital for modern switchable photonic and optoelectronic devices. The LC technology dominates in high-performance light modulators such as displays and active retarders. The next largest remaining technological barrier preventing further improvement of the liquid crystal display (LCD) technology is limited by viscoelastic properties relatively slow electro-optic response time of the LC molecules operating in dielectric coupling mode to applied electric field. The flexoelectro-optic effect allows improving of LCD’s response time down to a sub-millisecond range. Such a breakthrough would open up potential avenues for active fast-switching retarders and commercialization of color sequential displays significantly boosting the performance and energy efficiency of current LCD technology.

Flexoelectric properties of LCs have been a subject of extensive scientific investigations motivated by theoretical interest and potential applications^1^,^2^,^3^,^4^,^5^,^6^,^7^. The flexoelectric effect in LC materials describes splay and bend deformations of the LC director, **n**, describing an average orientation of molecules induced by applied external electric field, as follows^3^,^4^:


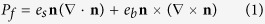


where *P*_*f*_ is flexoelectric polarization, *e*_*s*_ and *e*_*b*_ are the splay and bend flexoelectric coefficients. Flexoelectricity in a geometry of uniform lying helix (ULH) cholesteric liquid crystal (CLC) texture with helical axis parallel to the plane of substrate deforms helical structure of CLC producing macroscopic rotation of the helical axis (HA) in the plane of the LC cell^1^:


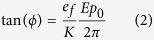


where *E*-applied electric field, *K*-effective elastic constant: *K* = (*K*_11_ + *K*_33_)/2, *K*_11_*, K*_33_-elastic constants for splay and bend deformation of LC director, *e*_*f*_ = (*e*_*s*_ + *e*_*b*_)/2–effective flexoelectric constant, and *p*_0_ is the equilibrium CLC helical pitch. A convenient value characterizing flexoelectric properties of a LC material is flexo-elastic coefficient *e*_*f*_/*K*^8^,^9^,^10^,^11^,^12^,^13^,^14^,^15^,^16^,^17^,^18^,^19^. Flexoelectric rotation of the HA always competes with undesired unwinding of the ULH CLC texture into a non-helical state due to dielectric coupling to applied electric field. Therefore, an ideal LC mixture for flexoelectro-optic switching would have as large as possible flexo-elastic coefficient and at the same time as small as possible absolute value of dielectric anisotropy.

A practical challenge in fabrication of a ULH flexoelectro-optic LC device is the process of obtaining and stabilization of a uniform single domain ULH CLC texture. The ULH texture is thermodynamically not stable with neither of uniform homeotropic nor planar alignment conditions and often requires supporting electric field. Therefore, the ULH requires special treatment to be generated. A number of experimental techniques for obtaining a well-aligned ULH texture have been reported. Most commonly the ULH is generated in cells with a uniform planar alignment layer as a result of a controlled rate cooling of CLC mixture from isotropic phase down to chiral nematic phase with small electric field applied across the cell gap^1^,^6^,^7^,^20^,^21^,^22^. Another method to generate a well-aligned ULH CLC texture is mechanical deformation or shearing of cell substrates with respect to each other with^5^,^8^,^10^,^11^,^23^ or without^2^,^24^ applied electric field. A few of experimental works have reported generation of a well-aligned ULH texture using a combination of controlled rate cooling of a CLC and shearing of cells substrates with applied electric field^12^,^13^,^16^,^17^. A well-aligned ULH CLC texture can be obtained by application of pulsed electric field with empirically optimized duration and magnitude to the sample cell^9^,^18^,^19^. Alternatively, a spontaneous formation of the ULH CLC texture have been demonstrated by treatment of the surface of substrates creating periodic micro-grooves^15^,^25^, periodic anchoring conditions^14^,^26^, controlling pretilt angle of LC molecules^27^, periodic polymer channels^28^ or polymer walls^29^.

A well-aligned ULH CLC texture provides a birefringent layer and flexoelectro-optic effect which enables control of transmittance of light through a pair of crossed polarizers. A typical sub-millisecond analogue response time creates a significant potential for application of the flexoelectro-optic switching mechanism of LC in numerous photonic and electro-optic devices^6^,^7^,^23^,^30^. However, relatively small values of flexo-elastic coefficient of traditional LC mixtures consisting of rod-like molecules until recently have been a significant barrier for commercialization of flexoelecro-optic devices^2^,^5^. A breakthrough large flexo-elastic coefficients were discovered in LC dimers consisting of two rigid molecular parts connected by a flexible hydrocarbon spacer^8^,^9^,^10^,^22^. Unfortunately, most of initially reported LC dimers with large flexoelectric coefficients has nematic phase at the temperature significantly larger than a room temperature^13^. Mixtures of dimers lowered the operating temperature, but at the same time suffered from increased viscosity significantly enlarging the switching time^12^. Recently, a large range tuning of helical pitch in CLC mixtures made of similar LC dimers and with an oblique helicoidal director configuration has been demonstrated to be useful for electrically tunable reflective optical devices covering range of optical spectrum from UV to near IR^31^,^32^,^33^.

Here, we describe in details a giant flexoelectro-optic behavior of LC dimer CB7CB. Experimentally measured angle of an in-plane rotation of HA in polymer stabilized (PS) ULH CLC texture demonstrates ~40 times larger effective flexo-elastic coefficient of CB7CB comparing to a reference commercial nematic LC material MLC-2048. Strong flexo-elastic coefficient allows using CB7CB for a variety of electro-optic applications as a main component or a dopant for increasing flexo-elastic coefficients of other host LC materials.

## Experimental Results

The ULH CLC texture was obtained in electro-optic cells made by two glass substrates with patterned 5 × 5 mm square ITO electrodes overlapped with each other enabling to apply electric field across the cell gap. The inner surfaces of cell substrates were coated with a thin PI2555 polyimide alignment layer for planar alignment, mechanically buffed and assembled into a cell with antiparallel rubbing directions. The cell gap thickness was set by particle spacers placed at the edges of a cell and verified by an interference method to be 2.3 μm. All electro-optical measurements were performed with 633 nm He:Ne laser as a light source.

The LC dimer CB7CB (1″,7″-bis(4-cyanobiphenyl-4′-yl)heptane) consists of two rigid cyanobiphenyl parts of the molecule which are connected with seven unit long flexible methylene spacer. Flexoelctric properties of a laboratory synthesized LC dimer CB7CB were compared with a reference commercial dual frequency nematic LC mixture MLC-2048 (Merck) with dielectric anisotropy ranging from Δε = +3.3 at 0.1 kHz to Δε = −3.1 at 50 kHz, and a crossover frequency of 15 kHz. Small positive dielectric anisotropy of CB7CB^34^,^35^ and of LC mixture MLC-2048 is an important benefit of these materials for operation in flexoelectro-optic switching mode. The CLC mixtures were obtained by mixing LC host materials with laboratory synthesized chiral dopant iso-(6OBA)_2_ (D-Glucitol,1,4:3,6-dianhydro-,2,5-bis[4-(hexyloxy)benzoate])^19^. Additionally, reactive monomer RM257 (Merck), and a small amount of photoinitiator Ir651 (Ciba) were added into CLC mixtures for polymer stabilization of a nonpermanent ULH CLC texture. The CB7CB based CLC mixture consisted of 93.19 wt.% of CB7CB, 3.40 wt.% of chiral dopant, 3.25 wt.% of RM257, 0.16 wt.% of Ir651, and had CLC pitch of 383 nm. The MLC-2048 based CLC mixture consisted of 90.29 wt.% of MLC-2048, 6.61 wt.% of chiral dopant, 2.97 wt.% of RM257, 0.13 wt.% of Ir651, and had CLC pitch of 297 nm. Values of CLC pitch were experimentally verified from observed edges of selective reflection band of CLC mixtures in thick cells with a good planar alignment.

**Figure 1:**
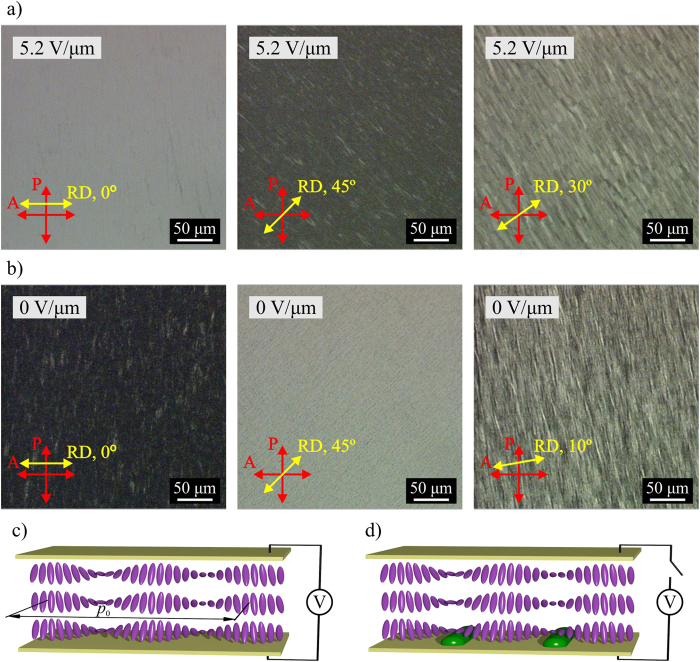
The POM micrographs of a well-aligned ULH texture with CB7CB CLC mixture with indicated rubbing direction (RD) of alignment layer: (**a**) nonpermanent ULH CLC texture before polymerization and with 5.2 V/μm applied; (**b**) the PS-ULH CLC texture without applied electric field. Schematic representations showing: (**c**) nonpermanent ULH CLC texture with supporting electric field, and (**d**) the PS-ULH CLC texture. Violet ellipses represent LC molecules, green half cylinders represent surface localized polymer.

Sample cells were filled with the CLC mixture in the isotropic phase and cooled down to a chiral nematic phase providing the Grandjean or a uniform standing helix texture. Afterwards, the ULH texture was generated at constant temperature by applying pulsed AC electric field to the sample gradually increasing the amplitude. If necessary, the sample was cycled a few times between higher and lower magnitudes of applied electric field until a well-aligned ULH texture was obtained. Typically, for tested CLC mixtures a 5.0–35.0 V/μm, 100–500 Hz square waveform bipolar AC electric field had to be applied to induce a nonpermanent ULH CLC texture. Compared CLC mixtures have ratio of cell gap thickness over equilibrium CLC pitch, *d/p*_0_ = 6 to 8. Using this technique we were able to obtain a well-aligned ULH texture of similar quality with all of CLC mixtures investigated in this work, as shown in Fig. 1.

The quality of obtained ULH CLC texture was visually monitored by observation of the sample between crossed polarizers using polarizing optical microscope (POM). Obtained the ULH CLC textures always had small amount of defects visible on POM micrographs as stripes with color different from the background caused by slightly imperfect alignment of the ULH texture. The ULH defects are especially easy to see if the HA is positioned in the orientation intermediate between 0° and 45° between crossed polarizers, as shown in Fig. 1. The best quality samples had electro-optic contrast ratio from flexoelectric switching in the range of 100:1 to 300:1.

Samples with nonpermanent ULH CLC texture were exposed to ultraviolet (UV) light to form surface localized polymer network and achieve stabilization of the ULH CLC texture, similarly to previously reported processing^5^,^6^,^7^,^19^. Photo-polymerization was performed using a UV light emitting diode (LED) light source (from UV ATA) with emission peak at ~365 nm, full width at half maximum (FWHM) of 9.2 nm, at operating temperature for each of CLC mixtures. The intensity of UV light for polymerization was 30 mW/cm^2^, and exposure time was 5 min. Electric field of minimum magnitude needed to maintain the nonpermanent ULH was kept applied across the ULH CLC layer during photo-polymerization. The appearance of nonpermanent ULH texture with applied supporting electric field and the PS-ULH after the polymerization at 0 V is shown in Fig. 1(a,b), respectively.

To confirm polymer stabilization and desired localization of polymer network on surfaces of cell substrates, processed PS-ULH CLC cells were opened, the LC was removed using *n*-hexane solvent, and the morphology of the polymer fibers was studied under a scanning electron microscope (SEM). The typical morphology of polymer fibers was observed to be different on top and bottom substrates.

**Figure 2:**
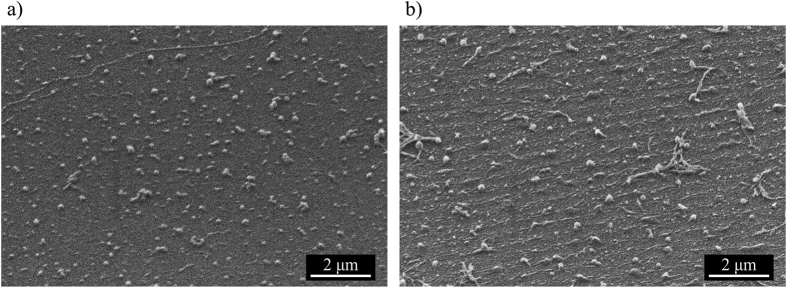
The 45° tilted SEM view showing polymer morphology in active electrode area with PS-ULH CLC texture on: (**a**) bottom substrate, and (**b**) top substrate.

The SEM images of the active electrode area with PS-ULH CLC texture on the bottom substrate which faces away from the UV light source during the polymerization show randomly distributed polymer aggregates with shape close to spherical and sometimes longer and narrower polymer fibers distributed on the surface without visible periodicity, as shown in Fig. 2(a). The size of polymer aggregates is typically in the range from ~50 nm to ~200 nm. On this substrate there is no visual difference of the polymer morphology inside and outside of electrode area.

The top substrate which faces UV light source during polymerization contains clearly visible periodic pattern formed in active electrode area with PS-ULH CLC texture as well as some of polymer particles similar to those observed on the bottom substrate, as shown in Fig. 2(b). The periodicity of long parallel polymer structures represents the half period of CLC helix. Sometimes, a typical CLC half-pitch dislocation defect can be distinguished in the periodic pattern. Periodic polymer pattern appears only in active electrode area containing the PS-ULH CLC texture. Outside of the electrode area the CLC is polymer stabilized with a Grandjean texture. Substrate area with the polymer stabilized Grandjean texture shows aggregations of randomly oriented polymer fibers possibly partially collapsed on the surface from the bulk of PS CLC layer together with randomly distributed polymer particles. The edge between the PS-ULH CLC and PS Grandjean textures is clearly distinguishable by the difference in morphology of surface localized polymer. The amount of polymer on the top substrate visually larger compared to the bottom substrate suggesting non-uniform distribution of polymer in the PS-ULH CLC layer.

**Figure 3:**
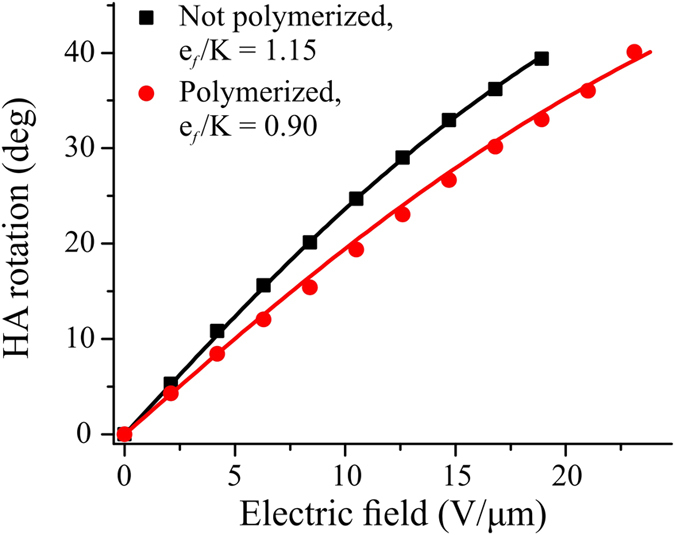
Electric field induced in-plane rotation of HA before and after photo-polymerization.

The flexoelectro-optic switching of PS-ULH CLC samples was characterized by a direct measurement of an in-plane rotation of HA and a static transmittance-voltage response (TV curve) of the sample to applied bipolar square wave AC electric field. Polymer network stabilizes nonpermanent ULH CLC texture and at the same time partially suppresses magnitude of flexoelectro-optic response. Flexoelectric in-plane rotation of HA as a function of applied electric field measured before and after photo-polymerization is shown in Fig. 3. The fitting of experimental data using eq. 2 demonstrates that polymer stabilization of the ULH CLC texture with 3.23 wt.% of polymer decreased the effective flexo-elastic coefficient of the LC mixture by 21.7%, from 1.15 C/N/m to 0.90 C/N/m. The LC host for the CLC mixture used for measurement shown in Fig. 3 consists of 59.8 wt.% of MLC 2048 and 40.2 wt.% of CB7CB, and the helical pitch of CLC mixture was 237 nm. Measurements before and after the polymerization were performed at room temperature (22 °C).

Due to the flexoelectric coupling to applied electric field the HA of ULH CLC texture rotates in the plane of the cell. Therefore, the flexoelectro-optic switching creates electric-field-induced transmittance through the sample between a pair of crossed polarizes. With the HA initially placed parallel to one of a pair of crossed polarizers (normally dark configuration), the maximum transmittance is expected at 45° rotation of the HA. The larger the effective flexoelastic coefficient of the CLC mixture, the smaller electric field is needed to achieve the maximum transmittance.

**Figure 4:**
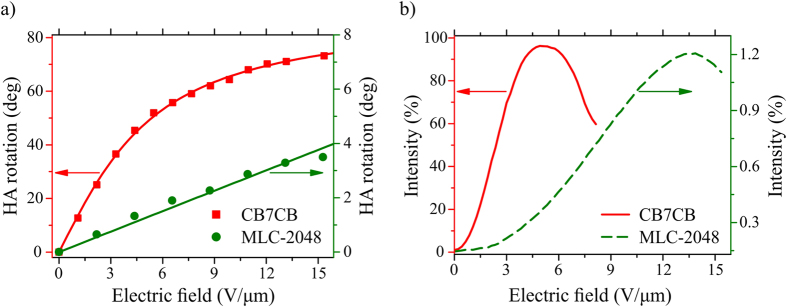
Flexoelectro-optic performance of CB7CB and MLC-2048 based PS-ULH CLC samples: (**a**) an in-plane rotation of HA as a function of applied electric field; (**b**) TV curves.

The HA rotation as a function of applied electric field in PS-ULH CLC samples is based on CB7CB and MLC-2048, and corresponding TV curves resulting from flexoelectric switching are compared and presented in Fig. 4. The PS-ULH CLC made of MLC-2048 has relatively small flexoelastic constant of 0.093 C/N/m providing the maximum measured HA rotation of 3.8°. In this CLC mixture the maximum of flexoelectric electric-field-induced transmittance was observed to be 1.2% at 13 V/μm. Further increase of applied electric field results in the beginning of dielectric unwinding of the PS-ULH texture and a gradual decrease of transmittance.

The PS-ULH sample consisting of CB7CB LC host has a flexo-elastic coefficient of 3.67 C/N/m. This value is ~13.6% larger than flexo-elastic coefficient of a similar odd-spaced LC dimer CB11CB with a four unit longer methylene spacer chain measured at reduced temperature −10 °C and without polymer stabilization^21^,^36^. The CLC mixture based on CB7CB provides the 45° of HA rotation at 4.5 V/μm, the maximum of electric-field-induced flexoelectric transmittance of 96.2%, and the maximum of measured HA rotation of 73.2° at 15.3 V/μm. The TV curves shown in Fig. 4(b) were measured in normally dark configuration of polarizers and normalized with respect to the transmittance of the same PS-ULH CLC sample at 0 V with HA aligned at 45° between crossed polarizers. Due to different temperature ranges of nematic LC phase measurements were performed at a room temperature (22 °C) for CLC mixture based on MLC-2048 and at 106 °C for CLC mixture based on CB7CB.

**Figure 5:**
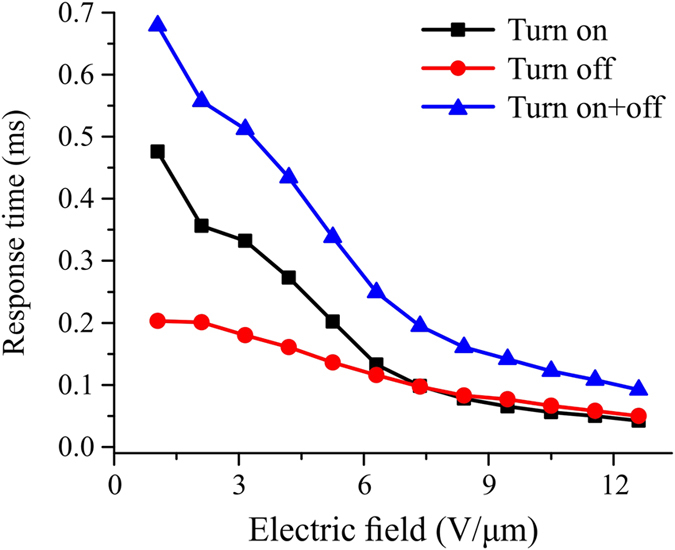
The flexoelectro-optic response time of CB7CB based PS-ULH CLC sample.

The flexoelectro-optic response time of CB7CB based CLC mixture is shown in Fig. 5. The response time was measured for switching between 0 V and V On state and calculated between 10% and 90% change of transmitted light intensity. The flexoelectro-optic response time clearly decreases as the magnitude of applied electric field is increased. In all of compared CLC mixtures the combined turn on and turn off flexoelectric response time is smaller than 1.0 ms.

## Conclusions

In conclusion, the giant flexo-elastic coefficient of LC dimer CB7CB was demonstrated with maximum of flexoelectrically-driven in-plane rotation of HA in PS-ULH CLC texture of 73.2°. Measured flexo-elastic ratio of CB7CB of 3.67 C/N/m is ~40 times larger than in a commercial LC mixture MLC-2048, and is the largest among reported in the literature up-to-date for an equivalent measurement technique. The 45° rotation of HA necessary for full intensity modulation of transmitted through light a pair of crossed polarizers is achieved with 4.5 V/μm. A sub-millisecond electro-optic switching time is demonstrated for all of compared CLC mixture compositions. Demonstrated giant flexoelectric properties of CB7CB evidence a significant potential for application of this LC dimer for ultra-fast switching flexoelectro-optic photonic and optoelectronic devices such as active retarders for quantum computing, new generation color sequential, and 3D LC displays.

## Additional Information

**How to cite this article**: Varanytsia, A. and Chien, L.-C. Giant Flexoelectro-optic Effect with Liquid Crystal Dimer CB7CB. *Sci. Rep.* **7**, 41333; doi: 10.1038/srep41333 (2017).

**Publisher's note:** Springer Nature remains neutral with regard to jurisdictional claims in published maps and institutional affiliations.
